# The epidemiology of psoriatic arthritis in Israel – a population-based study

**DOI:** 10.1186/s13075-017-1497-4

**Published:** 2018-01-02

**Authors:** Lihi Eder, Arnon Dov Cohen, Ilan Feldhamer, Sari Greenberg-Dotan, Erez Batat, Devy Zisman

**Affiliations:** 1 Women’s College Research Institute, Women’s College Hospital, Suite 6326, 76 Grenville St, Toronto, Ontario M5S 1B2 Canada; 2 Department of Medicine, University of Toronto, Toronto, ON Canada; 3 Chief Physician’s Office, Central Headquarters, Clalit Health Services, Tel Aviv, Israel; 4 Siaal Research Center for Family Medicine and Primary Care, Faculty of Health Sciences, Ben-Gurion University of the Negev, Be’er-Sheva, Israel; 5 Department of Rheumatology, Carmel Medical Center, Haifa, Israel; 6 The Ruth and Bruce Rappaport Faculty of Medicine, Technion—Israel Institute of Technology, Haifa, Israel

**Keywords:** Psoriasis, Psoriatic arthritis, Spondyloarthritis, Epidemiology, Middle East

## Abstract

**Background:**

There is limited information on the epidemiology of psoriatic arthritis (PsA) in general and in Middle Eastern populations in particular. The aims of this study were to estimate the prevalence and incidence rates of PsA and their temporal trends in the general population in Israel.

**Methods:**

In this study, a cohort of adult patients with PsA was derived from the database of Clalit Health Services (CHS), Israel’s largest health fund, with over 4.4 million members. The crude and age- and sex-standardized prevalence and incidence rates of PsA from 2006 to 2015 in the general population were calculated. The variation in PsA prevalence was assessed in relation to several demographic factors.

**Results:**

Among the 2,931,199 individuals aged 18 years and older registered in the CHS database in 2015, 4490 patients had a diagnosis of PsA (322 incident cases), resulting in overall crude prevalence and incidence rates of 0.153% (95% CI 0.149%, 0.158%) and 10.9 (95% CI 9.8, 12.3) per 100,000 population, respectively. The reported prevalence of PsA in Israel has doubled between 2006 and 2015 (from 0.073% to 0.153%). In contrast, the global incidence rate remained stable, with a gradual increase in incidence among individuals aged 51 to 70 years. PsA is associated with Jewish ethnicity, high socioeconomic status, and higher body mass index.

**Conclusions:**

The prevalence and incidence of PsA in Israel are within the range of previous estimates from Southern European populations. An increase in the reported prevalence of PsA was observed over the past decade in the general population in Israel.

## Background

Psoriasis is a chronic inflammatory skin condition affecting 2–3% of the general population in Europe and North America [1, 2]. Psoriatic arthritis (PsA) is a chronic, potentially destructive inflammatory arthritis affecting people with psoriasis. There is limited data about the population-based epidemiology of PsA. Researchers have estimated that the prevalence of PsA in the general population ranges from 0.05% to 0.67%, whereas the incidence rates range from 6.1 to 41.3 [3–7] per 100,000 population. The majority of these studies were conducted in Europe and North America among people of European origin. Researchers in recent studies in East Asia reported significantly lower prevalence and incidence rates of PsA among Asian individuals [8–10]. There is very little data about the epidemiology of PsA in Middle Eastern populations from various ethnic groups [11, 12]. Assessing the epidemiology of PsA in such populations could contribute to the understanding of the worldwide burden of the disease. Furthermore, because PsA susceptibility is strongly affected by environmental and genetic factors, studying the epidemiology of the disease in ethnically diverse populations from different geographic regions could potentially contribute to the understanding of underlying mechanisms of the disease. The aims of this study were to estimate the prevalence of PsA and its variation among different age, sex, and ethnic subgroups in the general population in Israel and to assess the temporal trends in the population-based prevalence and incidence of PsA in Israel over the past decade.

## Methods

### Patients and setting

The study was conducted using data from 2002 to 2015 stored in the Clalit Health Services (CHS) database. CHS is one of the four not-for-profit health maintenance organizations that provide hospital- and community-based medical services, including medical treatments, diagnostic tests, and hospitalizations, for the Israeli population. All Israeli residents are required by law to choose to be covered by one of the health maintenance organizations, of which CHS is the largest, providing medical services to approximately 4.4 million enrollees (52% of Israel’s population). The population served by CHS is broadly representative of the Israeli population in terms of demographics, geographic distribution, and ethnic diversity. The CHS database includes detailed demographics and clinical data, including sociodemographic information derived from Israel’s Central Bureau of Statistics and the National Insurance Institute (Social Security). The database has real-time input from pharmaceutical, medical, and administrative operating systems. The data are compiled into a centralized data warehouse from electronic medical records from primary care and specialist clinics, hospitals, pharmacies, and laboratories. CHS also coordinates a chronic disease registry, and all affiliated primary care physicians provide clinical information on all subjects aged > 20 years with any of 110 chronic diseases based on International Classification of Diseases, Ninth Revision (ICD-9), codes. The CHS database has been used in numerous epidemiological studies, including in the field of psoriatic disease [13, 14].

The study population included all subjects aged 18 years and older and enrolled in CHS at any point between 2006 and 2015. Patients were followed from the time of enrollment in CHS until death, their leaving CHS, or the end of the study in December 2015. The primary analysis of the global and subgroup prevalence of PsA was conducted using the entire CHS population of 2015. The temporal trends in the prevalence and incidence rates of PsA from 2006 to 2015 were assessed. For this analysis, the study populations included all CHS members aged 18 years and older for each assessed year. All data in this study were anonymous to the investigators. The study was approved by the research ethics board of Carmel Medical Center.

### Case definition of PsA and psoriasis

In a pilot study, an algorithm for identification of patients with PsA in the CHS database was validated [15]. Two rheumatologists reviewed the medical records of a random sample of 205 patients who received their initial diagnostic code of PsA (ICD-9 code 696.0) in 2014. Based on the medical chart review, a decision was made regarding the disease status: (1) definite/probable/possible PsA, (2) not PsA, or (3) insufficient/no data to verify diagnosis. Several candidate algorithms were tested to determine the one providing the highest positive predictive value. The selected algorithm comprised one of the following conditions: (1) PsA diagnosis assigned at least once by a rheumatologist; (2) permanent diagnosis code assigned by a primary care physician combined with use of synthetic or biologic disease-modifying antirheumatic drugs; or (3) PsA code listed in a hospitalization discharge summary. This algorithm had positive predictive value, sensitivity, and specificity of 90.5%, 88.7%, and 88.1%, respectively.

The methodology for identification of patients with psoriasis in the CHS database has been described previously [16–18]. Briefly, patients were defined as having psoriasis when there was at least one documented diagnosis of psoriasis in the medical records assigned by a CHS dermatologist or when psoriasis was listed in the diagnoses of hospital discharge letters.

### Additional information

The following variables were extracted from the database: age, sex, socioeconomic status (SES), ethnicity (Jewish or Arab), area of residence (urban vs. rural), and body mass index (BMI). SES was classified as low, middle, or high according to the neighborhood socioeconomic scores established by the Israel Central Bureau of Statistics as a proxy for SES, represented by a Z-score comparing the Israeli neighborhood mean SES with the neighborhood of a subject [19].

### Statistical analysis

The crude and age- and sex-standardized annual prevalence and incidence rates (with corresponding 95% CIs) were calculated for PsA by dividing the number of patients with PsA (aged 18 and over) by the total number of CHS enrollees (aged 18 and over) in the relevant year over the period 2006–2015. The CHS information is available from 2002; however, a 4-year “run-in” period was used to distinguish between incident and prevalent PsA cases. Thus, the prevalence and incidence rates are reported from 2006 onward. Disease onset is defined as the date of the first qualifying health services contact for which a diagnosis of PsA is provided. Patients with such first contacts were considered incident cases of PsA. Prevalent cases were carried forward from the first date they received the diagnosis of PsA until they died, left CHS, or the study ended (December 2015). Annual sex- and age-specific prevalence and incidence rates were calculated for 10-year age groups and expressed as proportions (95% CIs) using the 2006 Israeli population for direct age and sex standardization [20].

In addition, the crude prevalence of PsA (in 2015) was reported separately for each of the following subgroups: sex (males vs. females), ethnicity (Jewish vs. Arab), SES (high, moderate, low), area of residence (urban vs. rural), and BMI category (underweight, normal, overweight, obese). Information about model covariates was obtained for the year of 2015 from the CHS database. The first BMI recorded in the database in 2015 was used. SES was recorded at the end of the calendar year for all patients. Univariable and multivariable logistic regression analyses were conducted to assess the association between the above-detailed variables and PsA compared with the general population. Only patients with complete data were included in the analysis (27.9% of the subjects had missing BMI). A sensitivity analysis was conducted by removing BMI from the regression model. The results were expressed as ORs with 95% CIs.

## Results

Among the 2,931,199 individuals aged 18 years and older registered in the CHS database in 2015, 4490 patients had a diagnosis of PsA; of those, 322 patients had a new diagnosis of PsA, resulting in an overall crude prevalence rate of 0.153% (95% CI 0.149%, 0.158%) and incidence rate of 10.9 (95% CI 9.8, 12.3) per 100,000 population (Table 1). Additionally, 110,201 patients had a diagnosis of psoriasis, resulting in an overall crude prevalence of 3.759% (95% CI 3.738%, 3.781%). The prevalence of PsA among patients with psoriasis (in 2015) was 4.07%.

**Table 1:** Prevalence and incidence of psoriatic arthritis in Israel by age and sex in 2015

Age group (years)	Males			Females			All				
	PsA (*n*)	Total (N)	Prevalence (%)	PsA (*n*)	Total (N)	Prevalence (%)	PsA (*n*)	New PsA (N)	Total (N)	Prevalence (%)	Incidence^a^
18–30	73	370,656	0.019	129	389,179	0.033	202	34	759,835	0.027	4.5
31–40	275	306,169	0.089	286	303,310	0.094	561	50	609,479	0.092	8.2
41–50	348	208,302	0.167	359	210,432	0.171	707	62	418,734	0.169	14.8
51–60	482	188,410	0.255	548	209,250	0.262	1,030	79	397,660	0.259	19.9
61–70	569	178,702	0.318	674	201,982	0.333	1,243	60	380,684	0.326	15.8
71–80	225	94,914	0.237	278	121,381	0.229	503	27	216,295	0.232	12.5
>80	93	56,517	0.164	151	91,995	0.164	244	10	148,512	0.164	6.7
All	2065	1,403,670	0.147	2425	1,527,529	0.159	4490	322	2,931,119	0.153	10.9

*PsA* Psoriatic arthritis

^a^Per 100,000 population

**Fig. 1:**
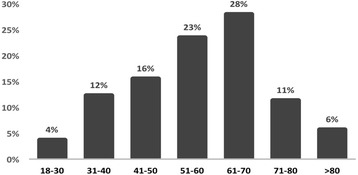
The distribution of age at diagnosis of psoriatic arthritis among all patients

The prevalence of PsA was slightly higher in females (0.159%; 54% of patients) than in males (0.147%; 46% of patients). The prevalence of PsA peaked in the seventh decade of life, at 0.327%. The age at diagnosis of PsA was higher than reported in previous cohorts from Europe and North America, because over half the patients were diagnosed after the age of 50 years (Fig. 1).

Most patients with PsA had a recorded diagnosis of psoriasis (3869 patients [86.2%]). In the majority (2371 [61.3%]), the diagnosis of PsA was recorded following the diagnosis of psoriasis. In 1061 patients (27.4%), the diagnoses of PsA and psoriasis were recorded in the same year, and in the remaining 11.3% of patients, the diagnosis of PsA preceded the diagnosis of psoriasis.

### Temporal trend in the prevalence and incidence of PsA from 2006 to 2015

**Fig. 2:**
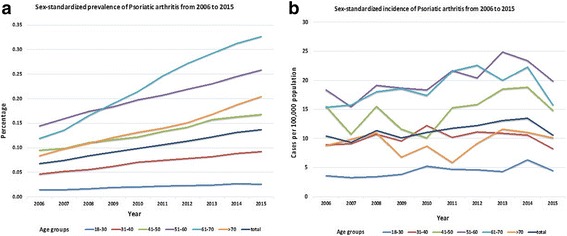
Sex-standardized prevalence (**a**) and incidence (**b**) of psoriatic arthritis in Israel by age group from 2006 to 2015

The crude prevalence of PsA doubled during the study period, from 0.073% in 2006 to 0.153% in 2015 (Table 2). A similar increase was observed in the age- and sex-standardized PsA prevalence: from 0.067% in 2006 to 0.137% in 2015 (204% increase). The crude and the age- and sex-standardized incidence rates remained stable from 2006 to 2015 with (approximately 11 per 100,000 population). The prevalence and incidence rates increased in similar degrees in both sexes over time (data not shown). The temporal trends in sex-standardized prevalence and incidence of PsA by age groups are shown in Fig. 2. A more pronounced increase in the prevalence of PsA was observed in the older age groups (61–70 and > 70 years). A gradual upward trend in the incidence rate was observed among middle-aged individuals (51–60, 61–70 years).

**Table 2:** Crude and age- and sex-standardized prevalence and incidence of psoriatic arthritis in Israel by year (2006–2015)

Year	Prevalence of PsA				Incidence of PsA		
	Total population (*N*)	PsA (*n*)	Crude prevalence (%)	Standardized prevalence (%) (95% CI)^a^	New PsA (*n*)	Crude incidence#	Standardized incidence^a^ (95% CI)^b^
2006	2,620,136	1910	0.073	0.067 (0.065, 0.070)	277	10.6	10.4 (9.5, 11.4)
2007	2,648,067	2132	0.081	0.075 (0.072, 0.077)	251	9.8	9.3 (8.4, 10.3)
2008	2,676,404	2425	0.091	0.084 (0.081, 0.086)	308	11.5	11.4 (10.4, 12.4)
2009	2,705,397	2663	0.098	0.091 (0.088, 0.093)	278	10.3	10.1 (9.2, 11.1)
2010	2,734,705	2938	0.107	0.099 (0.096, 0.102)	303	11.1	11.1 (10.1, 12.1)
2011	2,777,279	3237	0.117	0.107 (0.104, 0.110)	330	11.9	11.7 (10.8, 11.8)
2012	2,816,452	3531	0.125	0.114 (0.111, 0.117)	382	12.5	12.3 (11.3, 13.3)
2013	2,861,934	3866	0.135	0.122 (0.119, 0.125)	354	13.4	13.1 (12.0, 14.1)
2014	2,905,530	4223	0.145	0.131 (0.128, 0.134)	404	13.9	13.5 (12.5, 14.6)
2015	2,931,199	4490	0.153	0.137 (0.134, 0.141)	322	11.0	10.6 (9.7, 11.6)

*PsA* Psoriatic arthritis

^a^Age- and sex-standardized to Israel population in 2006

^b^Crude and standardized rates are per 100,000 population

### Factors associated with PsA

The prevalence of PsA was associated with several demographic and lifestyle factors, as shown in Table 3. The sex distribution of PsA was overall balanced. An association was found between the level of SES and PsA. PsA was most frequent in people with a higher SES (high SES prevalence 0.201%, adjusted OR high vs. low SES 1.29; 95% CI 1.19, 1.42), followed by middle SES (moderate SES prevalence 0.167%, OR middle vs. low SES 1.11; 95% CI 1.03, 1.20) and lowest in the low SES (prevalence 0.116%). Additionally, the distribution of PsA varied by ethnicity, with higher prevalence in individuals of Jewish ethnicity compared with Arabs (adjusted OR 1.64, 95% CI 1.47, 1.82). Last, in accordance with previous literature [21], PsA was more frequent in overweight (prevalence 0.201%; OR 1.41; 95% CI 1.30, 1.54) and obese individuals (prevalence 0.266%; OR 1.80; 95% CI 1.65, 1.97) than in people with normal weight (prevalence 0.104%). A sensitivity analysis that removed BMI from the regression model showed essentially the same results (data not shown).

**Table 3:** Prevalence of psoriatic arthritis by demographic characteristics (in 2015) and their association with psoriatic arthritis by logistic regression analysis

	Prevalence (%)	Univariable model		Multivariable model^a^	
		OR	95% CI	OR	95% CI
Sex					
Female	0.159	1.08	1.02, 1.15	1.03	0.96, 1.10
Male	0.147	1.00		1.00	
Socioeconomic status					
High	0.204	1.76	1.63, 1.90	1.29	1.19, 1.42
Middle	0.167	1.44	1.35, 1.55	1.11	1.03, 1.20
Low	0.116	1.00		1.00	
Ethnicity					
Jewish	0.175	2.13	1.95, 2.33	1.64	1.47, 1.82
Arab	0.082	1.00		1.00	
Residential area					
Urban	0.151	0.82	0.73, 0.92	1.02	0.89, 1.15
Rural	0.184	1.00		1.00	
BMI, kg/m^2^					
> 30	0.266	2.57	2.37, 2.80	1.80	1.65, 1.97
25–30	0.200	1.94	1.78, 2.10	1.41	1.30, 1.54
18.5–25	0.106	1.00		1.00	
< 18.5	0.068	0.63	0.47, 0.86	0.98	0.72, 1.33

*BMI* Body mass index

^a^The model was also adjusted for age group

*CI* Confidence Interval, *OR* Odds Ratio

## Discussion

In this population-based study, the temporal trends in the prevalence and incidence of PsA in Israel over the past decade were described. The following major findings are presented:

1. In 2015, the prevalence of PsA in the adult population in Israel was 0.153%, and the incidence rate was 10.9 per 100,000 population.
2. The reported prevalence of PsA in Israel has doubled over the past decade. In contrast, the global incidence rate remained relatively stable, except for a gradual increase in the rate among individuals aged 51 to 70 years.
3. PsA was more frequent in people from the following groups: Jewish ethnicity, higher SES, and higher BMI.

The population-based epidemiology of PsA has been investigated mostly in European populations. Limited information exists about the epidemiology of PsA in other ethnic groups and geographic regions, in particular in Middle Eastern populations. Researchers in a few small clinic-based studies assessed epidemiologic features of PsA in Iranian and Turkish patients [11, 12]. To the best our knowledge, this is the first large-scale population-based study that provides information about the prevalence of PsA in a Middle Eastern population including two ethnically diverse subpopulations. Our estimates of the prevalence of PsA in Israel (0.153%) fall well within the prevalence range reported in other population-based studies in Southern European countries, including Greece (0.06–0.17%) [22, 23] and Turkey (0.05%) [11], but tended to be lower than estimates in the United States (0.25%) [24] and Northern European countries such as Sweden (0.25%) [25], Norway (0.19–0.67%) [6, 26], the United Kingdom (0.19%) [3], and Iceland (0.16%) [27]. Moreover, the prevalence of PsA was significantly higher than the reported prevalence in East Asian populations, which is less than 0.10% [9, 10]. Fewer studies assessed the incidence of PsA. Overall, our estimated incidence rate of 10.9 cases per 100,000 population fell within previous estimates in other European populations, which ranged from 6.1 to 41.3 per 100,000 population [4, 6, 7]. The variation in prevalence rates may be attributed to genetic and environmental factors but also to variations in study designs and case definitions. In general, surveys that were based on self-reported diagnosis of PsA found higher prevalence rates than studies that relied on medical records and health administrative data.

The prevalence of PsA among patients with psoriasis in our study was 4.07%, which is lower than the estimated prevalence in other population-based studies (ranging from 4.6% to 11%) [3, 24, 28]. Possible explanations may include the low prevalence of the HLA-B27 antigen in the Israeli population [29], the older peak age of diagnosis of PsA observed in our cohort (range 60 to 70 years) [3, 26, 27], or possibly other genetic and environmental factors that are unique to our population.

We have demonstrated that the crude and age-adjusted prevalence of PsA doubled during the study period. These findings are in line with recent studies from Europe and Asia in which researchers reported an increase in the prevalence and incidence of psoriasis and PsA over time [7, 9, 30, 31]. The observed increase in prevalence may be partially attributed to an increase in the incidence rate among individuals aged 51 to 70 years, which is the peak age of diagnosis of PsA in Israel. Increased awareness of PsA among physicians over the past decade, possibly promoted by the publication of the Classification Criteria for Psoriatic Arthritis (CASPAR) criteria in 2006, educational activities among specialists, the introduction of effective biologic medications for PsA, and the increased use of sensitive diagnostic modalities (e.g., ultrasound) likely contributed to the observed time period effect. Additionally, with the increasing awareness of the disease, it is possible that rheumatologists are more likely to classify a disease as “PsA” rather than “spondyloarthritis,” which may explain some of the increase in occurrence over the past decade. Other potential causes, such as increased occurrence of known risk factors for PsA (e.g., obesity) at the population level or changes in the population demographics (e.g., immigration of high-risk groups), are less likely to contribute to the observed increase over such a short time period.

Several demographic factors were associated with the occurrence of PsA. As in other studies, PsA was almost equally distributed between women and men (female/male ratio 1.08) [3]. Other factors that influenced the occurrence of PsA were ethnicity, SES, and BMI. The prevalence of PsA was almost twofold higher in the Jewish population than among Arab individuals. This difference in disease occurrence may be related to variations in genetic structure, environmental exposures, or issues related to healthcare use [21]. With regard to impact of genetic heterogeneity within the Jewish population on the occurrence of PsA, information about family origin is unavailable in the CHS database.

This study has several limitations that should be taken into consideration. First, we could not capture patients with PsA who did not seek medical attention or those who were undiagnosed as having PsA. Nonetheless, we estimated the proportion of the population that places a burden on the healthcare system. Second, the case definition is based on diagnostic coding from electronic medical records that are based on physician diagnoses. This may have led to misclassification of cases. This risk, however, was minimized by selection of an algorithm with high accuracy that used data based largely on a diagnosis by relevant specialists. The primary strength of the study is the use of a large representative sample of over half the Israeli population. Consequently, the results of the study could be generalized to the broader population beyond those patients included in our study.

## Conclusions

In summary, the epidemiology of PsA is much less studied than in many other rheumatic diseases. High-quality real-world data about the epidemiology of PsA is of clear importance for understanding the burden of the disease on society. Our study, which included over 4000 patients with PsA, is one of the largest population-based studies to date and is the first to include an evaluation of the prevalence of PsA in Middle Eastern individuals. The prevalence of PsA in the adult Israeli population in 2015 was 0.153%. The reported prevalence of PsA has doubled over the past decade, highlighting the rising medical needs and economic burden of this patient population. Further studies are needed to continue monitoring this trend and understand its underlying causes.

## Abbreviations

BMI: Body mass index
CHS: Clalit Health Services
PsA: Psoriatic arthritis
SES: Socioeconomic status

## References

[1] Gelfand JM, Weinstein R, Porter SB, Neimann AL, Berlin JA, Margolis DJ (2005). Prevalence and treatment of psoriasis in the United Kingdom: a population-based study. Arch Dermatol.

[2] Langley RG, Krueger GG, Griffiths CE (2005). Psoriasis: epidemiology, clinical features, and quality of life. Ann Rheum Dis.

[3] Ogdie A, Langan S, Love T, Haynes K, Shin D, Seminara N (2013). Prevalence and treatment patterns of psoriatic arthritis in the UK. Rheumatology (Oxford).

[4] Alamanos Y, Voulgari P, Drosos A (2008). Incidence and prevalence of psoriatic arthritis: a systematic review. J Rheumatol.

[5] Stolwijk C, van Onna M, Boonen A, van Tubergen A (2016). Global prevalence of spondyloarthritis: a systematic review and meta-regression analysis. Arthritis Care Res (Hoboken).

[6] Hoff M, Gulati AM, Romundstad PR, Kavanaugh A, Haugeberg G (2015). Prevalence and incidence rates of psoriatic arthritis in central Norway: data from the Nord-Trondelag health study (HUNT). Ann Rheum Dis.

[7] Egeberg A, Kristensen LE, Thyssen JP, Gislason GH, Gottlieb AB, Coates LC (2017). Incidence and prevalence of psoriatic arthritis in Denmark: a nationwide register linkage study. Ann Rheum Dis.

[8] Oh EH, Ro YS, Kim JE (2017). Epidemiology and cardiovascular comorbidities in patients with psoriasis: a Korean nationwide population-based cohort study. J Dermatol.

[9] Wang TS, Hsieh CF, Tsai TF (2016). Epidemiology of psoriatic disease and current treatment patterns from 2003 to 2013: a nationwide, population-based observational study in Taiwan. J Dermatol Sci.

[10] Li R, Sun J, Ren LM, Wang HY, Liu WH, Zhang XW (2012). Epidemiology of eight common rheumatic diseases in China: a large-scale cross-sectional survey in Beijing. Rheumatology (Oxford).

[11] Cakir N, Pamuk ON, Dervis E, Imeryuz N, Uslu H, Benian O (2012). The prevalences of some rheumatic diseases in western Turkey: Havsa study. Rheumatol Int.

[12] Jamshidi F, Bouzari N, Seirafi H, Farnaghi F, Firooz A (2008). The prevalence of psoriatic arthritis in psoriatic patients in Tehran. Iran Arch Iran Med.

[13] Shapiro J, Cohen AD, Weitzman D, Tal R, David M (2012). Psoriasis and cardiovascular risk factors: a case-control study on inpatients comparing psoriasis to dermatitis. J Am Acad Dermatol.

[14] Zisman D, Bitterman H, Shalom G, Feldhamer I, Comanesther D, Batat E (2016). Psoriatic arthritis treatment and the risk of herpes zoster. Ann Rheum Dis.

[15] Eder L, Cohen AD, Feldhamer I, Greenberg-Dotan S, Batat E, Zisman D. Validity of diagnostic codes and point prevalence of psoriatic arthritis in Israel – a population-based study [abstract 1697]. Arthritis Rheumatol. 2016;68(Suppl 10).

[16] Cohen AD, Sherf M, Vidavsky L, Vardy DA, Shapiro J, Meyerovitch J (2008). Association between psoriasis and the metabolic syndrome: a cross-sectional study. Dermatology.

[17] Cohen AD, Dreiher J, Shapiro Y, Vidavsky L, Vardy DA, Davidovici B (2008). Psoriasis and diabetes: a population-based cross-sectional study. J Eur Acad Dermatol Venereol.

[18] Dreiher J, Weitzman D, Shapiro J, Davidovici B, Cohen AD (2008). Psoriasis and chronic obstructive pulmonary disease: a case-control study. Br J Dermatol.

[19] Filc D, Davidovich N, Novack L, Balicer RD (2014). Is socioeconomic status associated with utilization of health care services in a single-payer universal health care system?. Int J Equity Health.

[20] Statistical Abstract of Israel 2006. Israeli Central Bureau of Statistics. http://www.cbs.gov.il/reader/shnaton/shnatonh_new.htm?CYear=2006&Vol=57&CSubject=2. Accessed 22 Jan 2017.

[21] Love TJ, Zhu Y, Zhang Y, Wall-Burns L, Ogdie A, Gelfand JM (2012). Obesity and the risk of psoriatic arthritis: a population-based study. Ann Rheum Dis.

[22] Trontzas P, Andrianakos A, Miyakis S, Pantelidou K, Vafiadou E, Garantziotou V (2005). Seronegative spondyloarthropathies in Greece: a population-based study of prevalence, clinical pattern, and management. The ESORDIG study Clin Rheumatol.

[23] Alamanos Y, Papadopoulos NG, Voulgari PV, Siozos C, Psychos DN, Tympanidou M (2003). Epidemiology of psoriatic arthritis in northwest Greece, 1982-2001. J Rheumatol.

[24] Gelfand JM, Gladman DD, Mease PJ, Smith N, Margolis DJ, Nijsten T (2005). Epidemiology of psoriatic arthritis in the population of the United States. J Am Acad Dermatol.

[25] Haglund E, Bremander AB, Petersson IF, Strombeck B, Bergman S, Jacobsson LT (2011). Prevalence of spondyloarthritis and its subtypes in southern Sweden. Ann Rheum Dis.

[26] Madland TM, Apalset EM, Johannessen AE, Rossebo B, Brun JG (2005). Prevalence, disease manifestations, and treatment of psoriatic arthritis in Western Norway. J Rheumatol.

[27] Love TJ, Gudbjornsson B, Gudjonsson JE, Valdimarsson H (2007). Psoriatic arthritis in Reykjavik, Iceland: prevalence, demographics, and disease course. J Rheumatol.

[28] Karreman MC, Weel AE, van der Ven M, Vis M, Tchetverikov I, Nijsten TE (2016). Prevalence of psoriatic arthritis in primary care patients with psoriasis. Arthritis Rheumatol.

[29] Elkayam O, Segal R, Caspi D (2004). Human leukocyte antigen distribution in Israeli patients with psoriatic arthritis. Rheumatol Int.

[30] Danielsen K, Olsen AO, Wilsgaard T, Furberg AS (2013). Is the prevalence of psoriasis increasing? A 30-year follow-up of a population-based cohort. Br J Dermatol.

[31] Icen M, Crowson CS, McEvoy MT, Dann FJ, Gabriel SE, Maradit KH (2009). Trends in incidence of adult-onset psoriasis over three decades: a population-based study. J Am Acad Dermatol.

